# The evolution of trauma care in the Netherlands over 20 years

**DOI:** 10.1007/s00068-019-01273-4

**Published:** 2019-11-23

**Authors:** Falco Hietbrink, Roderick M. Houwert, Karlijn J. P. van Wessem, Rogier K. J. Simmermacher, Geertje A. M. Govaert, Mirjam B. de Jong, Ivar G. J. de Bruin, Johan de Graaf, Loek P. H. Leenen

**Affiliations:** Department of Surgery, University Medical Centre Utrecht, Heidelberglaan 100, 3584 CX Utrecht, The Netherlands

**Keywords:** Trauma systems, Mortality, Outcome analysis, Centralisation

## Abstract

**Introduction:**

In 1999 an inclusive trauma system was initiated in the Netherlands and a nationwide trauma registry, including all admitted trauma patients to every hospital, was started. The Dutch trauma system is run by trauma surgeons who treat both the truncal (visceral) and extremity injuries (fractures).

**Materials and Methods:**

In this comprehensive review based on previous published studies, data over the past 20 years from the central region of the Netherlands (Utrecht) was evaluated.

**Results:**

It is demonstrated that the initiation of the trauma systems and the governance by the trauma surgeons led to a region-wide mortality reduction of 50% and a mortality reduction for the most severely injured of 75% in the level 1 trauma centre. Furthermore, major improvements were found in terms of efficiency, demonstrating the quality of the current system and its constructs such as the type of surgeon. Due to the major reduction in mortality over the past few years, the emphasis of trauma care evaluation shifts towards functional outcome of severely injured patients. For the upcoming years, centralisation of severely injured patients should also aim at the balance between skills in primary resuscitation and surgical stabilization versus longitudinal surgical involvement.

**Conclusion:**

Further centralisation to a limited number of level 1 trauma centres in the Netherlands is necessary to consolidate experience and knowledge for the trauma surgeon. The future trauma surgeon, as specialist for injured patients, should be able to provide the vast majority of trauma care in this system. For the remaining part, intramural, regional and national collaboration is essential

## Introduction

Assessment, resuscitation and treatment of injured patients have seen major changes over the past 20 years in the Netherlands. The improvements that derived from these developments, in terms of decreased in-hospital mortality and morbidity, are mainly attributed to two factors. First of all, changes in the organizational structure of the trauma system were made. Second, trauma is nowadays more handled as an unique disease entity. The current system is, as in most countries, build on the holistic character of the trauma surgeon as a specialist for these patients. The data in this review paper are extracted from previous publications involving the trauma network “Midden Nederland”, which comprises the central region of the Netherlands and is made possible due to continuous prospective data gathering and outcome analysis.

## The Dutch system

All over the world, the (trauma) surgeon treats (severely) injured patients. In many countries, such as the USA and the Scandinavian countries, the emphasis is put on the most lethal truncal injuries. In these countries, the visceral surgeon is in charge of trauma care. In other countries, such as the German-speaking countries, a more quantitative approach is taken and the orthopaedic surgeon is nowadays in charge of trauma care, as over 80% of the surgical procedures in injured patients concerns extremity (fracture) surgery[1].

In the Netherlands, the trauma surgeon is a general surgeon with trauma-orthopaedic competences. The differentiation from general all-round surgeon towards trauma surgery started in the eighties and came to its current form in the early nineties. Nowadays, the Dutch trauma surgeon follows 4 years of training in general surgery and thereafter trains another 2 more years mainly in trauma surgery. In these last 2 years, additional training and courses in resuscitation, visceral and fracture surgery are required (ATLS refresher, DSTC, AO advanced). Additional fellowships are available to acquire specific competences when needed. Thereafter, trauma surgeons update their skills by a variety of courses and additional training in the wet-lab or cadaver training.

As a result, in the Netherlands the trauma surgeon treats both visceral injuries (neck/chest/abdomen/pelvis) and extremity injuries (including soft tissue injuries and fracture treatment) [2, 3]. In addition, the trauma surgeon in the Netherlands has knowledge of physiological disturbances and cares for the resuscitation of the patient in the acute phase [4, 5]. Thereafter, the surgeon is actively involved in the intensive care in our level 1 centre. This requires a broad spectrum of skills, based on solid education and a reliable training process.

This skill set enables the Dutch trauma surgeon to be involved with their patients in a longitudinal aspect during resuscitation and treatment [6, 7]. This process starts in the emergency department (ED) as the trauma surgeon in our level 1 trauma centre attends every trauma team activation (following high-energy mechanism supplemented with, but not solely depending on, vital parameters or stability). Thereafter, treatment continues through the operating room (OR) or intensive care (ICU), to the trauma ward and finally to the rehabilitation clinic if necessary. In the period after discharge, the trauma surgeon is also the primary person of contact in the out-patient clinics. There are, however, some local differences in the involvement of the trauma surgeons. For instance, in our hospital spine surgery is performed by a combination team of orthopaedic and neurosurgeons. Furthermore, prosthesis is placed by orthopaedic surgeons only, in contrast to other Dutch hospitals where other choices are made. Nevertheless, the generalistic background and holistic view of the Dutch trauma surgeon and specific dedication to trauma optimizes the integral treatment of injured patients. Furthermore, with the combination of both truncal and extremity trauma, there is sufficient volume to employ fulltime trauma surgeons without the need for a second specialty in a more elective or non-trauma setting [8]. In recent years, however, this generalistic character is challenged in an environment of public opinion and far-reaching legislation that demands ongoing sub-specialisation of physicians.

## The evolution of mortality in trauma

In 1999 the Netherlands implemented an inclusive trauma system, which organized the country in 11 trauma regions [9]. The goal of this inclusive system is to present all patients on a timely basis at the right hospital, with the centralisation of the most severely injured patients in level 1 centres [10-23]. The less severely injured patients are ideally treated at level 2 and level 3 centres. The composition of our region is described in Table 1, including a short description of the designated trauma level.

**Table 1:** General description of trauma centre level layout in the Netherlands

Trauma center	Function	Number in our region
Level 1	For the most severely injured patients, multitrauma patients and patients with brain injury. Fully equipped trauma center with twenty-for-seven open ER, helicopter landing pad, neurosurgical availability, immediate CT-scanning and angio-suit available and OR available < 15 min	1
Level 2	For patients with isolated or multiple injuries. Not for multitrauma patients or patients with brain injuries	3
Level 3	For patients with isolated injuries	2

This organizational change had a major impact on trauma care, as it did in most countries that implemented inclusive trauma systems [24, 25]. The data presented in every time period is based on previous publications and where possible adjusted odd ratios presented in those publications were used [10-23]. Before centralisation (step 1) a regional mortality rate of 2.6% was documented, of which 40% died due to exsanguination. Before 1999 the exsanguination percentage in the academic teaching hospital (the University Medical Centre Utrecht, later to be the level 1 trauma centre) was 17%. After centralisation (period 2003–2005) a concentration of severely injured (multitrauma) patients was noted (multitrauma defined as ISS > 15). This increase in trauma severity was accompanied by an increase in crude mortality (Table 2). However, when corrected for age and injury severity [based on the Injury Severity Score (ISS)], a reduction in odds ratio was found for mortality. This reduction in mortality could be attributed to a decrease in death due to exsanguination and organ failure. In the years thereafter, a further maturation of the trauma system took place (step 2).

**Table 2:** Number of patients and mortality per time period

Time period	1996–1998	2003–2005	2006–2009	2014–2016
Hallmark	Before centralization	After centralization	Optimizing trauma	Mind-set trauma
Injury Severity Score (ISS) in level 1 trauma centre (mean)	9.6	12.4	13.8	12.4
Number of total admitted injured patients in level 1 trauma centre (*n*/year; mean)	1401	1193	863	1348
Multitrauma patients in level 1 trauma centre (*n*/year; mean)^a^	156	186	225	358
Mortality in trauma region^b^	2.6%	2.3%	NA	1.2%
Odds ratio (OR) regional to previous period (corrected for age and ISS)	Reference	0.84	NA	NA
Mortality in level 1 trauma centre^b^	7.9%	8.5%	8.2%	5.2%
Odds ratio (OR) level 1 trauma centre to previous period (corrected for age and ISS)	Reference	0.61	0.74	0.54
Cause of mortality in level 1 centre				
Exsanguination	17%	9%	8%	3%
Organ failure	25%	18%	5%	2%
Neurological injuries	40%	57%	68%	85%

Data in this table were extracted from the previously published articles. The cited articles were based on the prospective database from the trauma region

*NA* not available

^a^Multitrauma was defined as an injury severity score (ISS) > 15

^b^The regional mortality rate is based on all trauma-related admissions. Similar, the mortality rate of the level 1 center is based on all patients admitted through the emergency department of that hospital

Parallel to the logistical optimization, there was a change in the surgical approach of severely injured patients. In conjunction to damage control surgery, the damage control principles are more frequently applied not only for the visceral surgery part but also in the resuscitation process (damage control resuscitation) and in the treatment of fracture-related injuries (damage control orthopaedics) [26-28]. Furthermore, non-operative management approaches are more widely acknowledged and appreciated. Moulded by education, training and positive results after the implemented modifications the mind-set of the trauma surgeon changed (step 3). The acute surgical patient and the trauma patient in particular is more and more being treated differently in comparison to the patient seen in the elective process. Trauma is regarded as a different entity, an unique disease. Decision-making, indication, timing and technique are all tailored to these patients. In the latest period (2014–2016), a gradual increase in the number of multitrauma patients was observed to over 350 annually, with an average ISS of 25 and a crude mortality of 14%. The calculated result is a further reduction in mortality compared to the previous period of almost 50%. This can not only be attributed to improved care immediately at presentation. Also for instance, by means of the 24 × 7 in house presence of a trauma surgeon it is attempted to further reduce the ‘failure-to-rescue’ rate in our centre [19]. These combined measures resulted that to date, the most important cause of death is neurotrauma (> 80%). Even more, in most cases of extensive neurotrauma it is a medical decision to stop treatment after adequate resuscitation and when a complete overview of the patients history and comorbidities is retrieved [16, 17]. Finally, next to the improved mortality rates, the care for trauma patients becomes increasingly efficient. Currently, the average length of stay in the hospital is 7 days, compared to 14 days before centralization. The length of stay at the ICU demonstrated a similar pattern with a reduction of 8–5 days in the same period.

This process is not confined to the level 1 trauma centre, but our whole trauma region (Utrecht) is part of this development. On the one hand, there is concentration of multitrauma patients and complex monotrauma patients (i.e. after high-energy mechanism, open fractures, patients with multiple comorbidities) in the level 1 centre. These patients require a multidisciplinary approach and benefit from physicians with experience in this pathology. On the other hand, there is concentration of patients with a single injury in the regional hospitals, who developed specific-efficient-patient pathways for these patient populations. This lateralisation (step 4) led to a reduction in mortality and more efficient care in for instance the geriatric patient with a hip fracture [22].

In conclusion, over the past 20 years the incremental steps and changes resulted in a regional reduction of 50% in crude mortality and a reduction in mortality adjusted for age and ISS of 75% in our level 1 trauma centre.

## A shift in outcome measurements: from mortality to functionality

To date, our level 1 trauma centre has a 5% mortality rate for trauma patients, with neurotrauma accounting for most of the fatalities. Due to the major reduction in mortality over the past few years, the opportunity arises to shift focus to the functional outcome of these patients. This is a significant change in perspective. However, especially in the multitrauma patient, this outcome can be difficult to determine. In consequence of the nature of the injuries, multitrauma patients are frequently excluded from functional outcome studies in a general trauma population, as these often severely injured patients have a “negative” impact on the study results. Negative results could be undesirable, as functional outcome studies are not only used to measure patient-reported outcomes, but also more and more to judge the quality of the care. As a result, little is known about the functional recovery and patient-reported outcomes of multitrauma patients. Recent results, however, have demonstrated that multitrauma patients can have a satisfying functional outcome and quality of life as well [18]. Furthermore, ṣover 90% of the patients who survived their neurotrauma eventually went home or to a rehabilitation centre. Of this latter group > 70% recovers to an acceptable level [17]. This also holds true for instance for patients with specific injuries like rib fractures, hand- or wrist injuries or complex foot injuries [29-31].

## Further centralisation of trauma care?

In the Netherlands, over 4000 multitrauma patients a year are admitted to any hospital. Current legislation demands that > 90% of the multitrauma patients are presented to and treated in a level 1 trauma centre. However, with this rate in 2017 being 53–78% in the different trauma regions of the Netherlands, this criterion has not yet been met [32]. One of the factors that hampers this triage rate is that the ISS is a post-assessment score. With the current questionable definition of the most severely injured patients (multitrauma or ISS > 16) and the lack of field triage criteria to adequately predict this definition, to us this goal of 90% seems unreachable [32]. The discussion what the ideal definition of multitrauma should be is ongoing, but currently the ISS > 16 is the one still used in the Netherlands. Furthermore, new tools are to be developed to improve field triage, but secondary transfers will remain needed to get all the patients in the correct level of care, both to and from level 1 [33]. To stimulate this process, government, health insurance companies and hospital boards should make active policy on this subject [34].

**Fig. 1:**
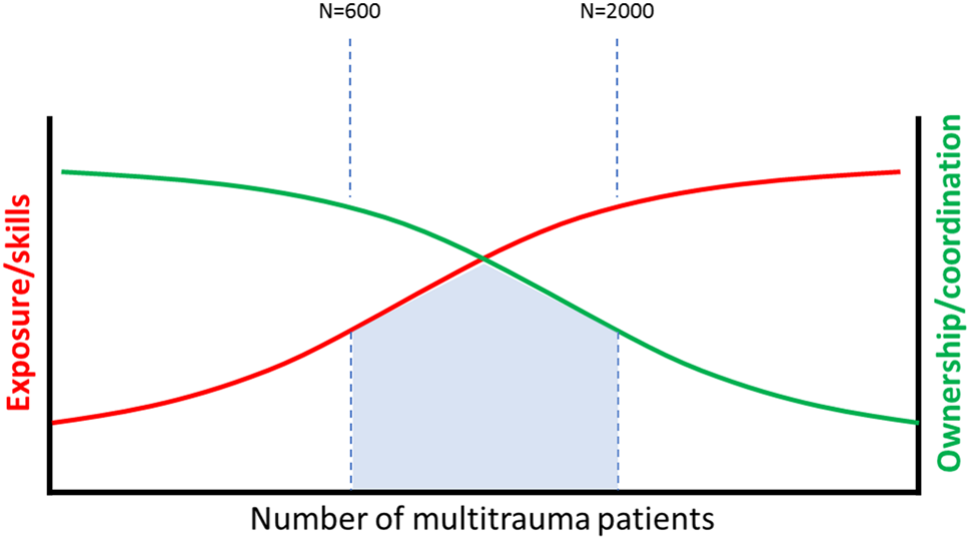
The effect of patient numbers. In case of too low numbers of severely injured patients, insufficient expertise per centre will be available to reduce mortality rates and optimize the logistic process. However, when the number of patients is too high patient ownership and coordination is hampered. It is likely that an optimum for the number of patients per centre exists

The optimal number of multitrauma patients per level 1 centre is much debated, but a plateau phase is suggested in the level of competence, knowledge and skills of the trauma team at approximately 600 of these patients per year [35, 36]. With this amount of severely injured patients logistical optimization and hospital efficacy also peaks. Nevertheless, bigger is not always better. In the largest centres of the world, with over > 2000 severely injured patients per year, the primary resuscitation and surgical stabilization are generally organized at an outstanding level. However, after stabilizing these patients (24–48 h later) treatment is easily scattered and coordination and follow-up may become difficult or even fully lost. As a result, longitudinal involvement of the trauma surgeon is challenging to achieve. Thus, in this perspective, it is reasonable to assume that, at least in our country, there might be an optimum of multitrauma patients per level 1 centre to strive for (Fig. 1) [35, 36]. For this, a larger catchment area per level 1 centre is probably necessary, and more frequent transport by air required. Whether these kind of numbers for further centralisation are achievable in the European and more specifically Dutch setting depends on the political ambitions and (supra-)regional agreements of all parties involved.

## Keeping the trauma surgeon alive?

It can be concluded that further centralization to a limited number of level 1 trauma centres is essential to consolidate experience and knowledge for the trauma surgeon. However, the pitfall of centralisation is an overshoot in sub-specializations, as level 1 centres are often aligned with top referent and academic centres with inherent sub-specialisation for elective cases. Although it would be possible to go down this road of sub-specialisation in trauma with a sufficient number of patients, we feel that it is undesirable. The added value of the trauma surgeon is longitudinal involvement in the care of injured patients and the broad, holistic scope of knowledge. Especially in the case of multiple injuries, the whole (the patient) is greater than the sum of its parts (separate injuries) [37]. Continuity of care and an integral treatment approach are essential for an optimal outcome [7].

The outcome in terms of mortality and functional recovery is mainly determined by timely diagnosis of injuries, the correct indication and timing of subsequent surgical procedures and the organisation of rehabilitation, in a continuous and aligned patient journey. As a consequence, someone has to be in charge of the process. This physician should be able to make a delicate decision between the different treatment strategies, both surgical and non-surgical, with knowledge of physiology, injuries and implications of treatment options and subsequent choices (Table 3). Physicians who are not actively involved in the surgical process will have a hard time balancing the decision, with a chance of less optimal outcome.

**Table 3:** Expertise and involvement with trauma per specialty

	Acute care dedication	Surgical specialty	Physiologic knowledge (trauma specific)	Long term care	Multisystem approach
Emergency physicians	++	−	+	−	++
Anaesthesiologists	+/−	−	++	−	±
Intensivists	+/−	−	+	−	+
Orthopaedic surgeons	−	++	−	++	−
Abdominal surgeons	−	++	+/−	++	−
Thoracic surgeons	−	++	−	−	−
Trauma surgeons	++	++	+	++	++

The challenge for the upcoming years will be to maintain the combination of competences, which can be depicted in model nowadays termed ‘neo-generalist’. This ‘tree-shape’ comprises of a broad foundation of knowledge and skills in multiple aspects of general surgery and basic physiology. In addition, the branching lines in the model indicates the competences for specialisation, in this case trauma. As stated, due to the low mortality rates in recent years, focus shifts from surviving to functional recovery. The biggest threat of this shift to improved functional outcome, as mentioned earlier, is a far-reaching sub-specialisation and the loss of the trauma surgeon as a broad developed specialist. It was previously demonstrated that there is a clear correlation between specialised trauma surgery training and the level of trauma system development [38]. It is likely that far-reaching sub-specialisation will come at the cost of success in reducing mortality. In the Netherlands, similar problems are encountered and described by Cardiologists and Psychiatrists in recent years [39-43].

The problem is that far-reaching sub-specialisation will lead to the disintegration of interrelated care and loss of focus of the patient as a whole. A balance should be sought between the width and depth of the expertise and skillset of the trauma surgeon. For this, it is supportive if trauma is seen as a disease entity and not as part of general duties when one is on call. In countries where the orthopaedic surgeon is in charge of trauma care, it can be challenging to provide integral lifesaving (damage control) surgery, especially when it concerns visceral injuries. On the other hand, in countries where the visceral surgeon is in charge of trauma care, a major part of injuries (and patients) is treated outside their scope as these concern only extremity injuries. This could result in a relative large number of missed or delayed diagnosed injuries [44, 45]. Additionally, when care is provided by a multitude of subspecialists, costs will rise extensively without a proven benefit for patients outcomes as stated previously [6]. It might be, that different countries choose different solutions for this rising problem. Nevertheless, in the inclusive trauma system, we have the opportunity to tune the competences of the surgeons to the needs of their patients in the different participating centres. Regardless of the centre and its position in the system, the injured patient needs a guide for longitudinal care, from presentation to rehabilitation.

In conclusion, there is good quality of care for the injured patient in the Netherlands, at the same time still room for improvement in the current cost-effective system. The goal for the upcoming years is to further centralise and differentiate in terms of function (expertise and organization) for the different levels of trauma centres. With this an even further reduction in mortality might be possible, while simultaneously aiming to improve functional outcome. The trauma surgeon as a specialist for injured patients is key in this model, being able to provide the vast majority of care, while for the remaining part, intramural, regional and national collaboration is essential.
